# Correspondence: Measurement of erythrocyte transketolase activity

**DOI:** 10.1155/S1463924683000292

**Published:** 1983

**Authors:** Donald E. King, C. R. Milner, J. E. Buttery, B. R. Chamberlain

**Affiliations:** Department of Clinical Chemistry The Queen Elizabeth Hospital Woodville South Australia 5011 Australia


Journal of Automatic Chemistry, Volume 5, Number 2 (April–June 1983), page 111

### Correspondence

### Measurement of erythrocyte transketolase activity

*Correspondence from Donald E. King*

C. R. Milner *et al.*'s recent paper compared automated versus manual procedures for the 'Measurement of erythrocyte transketolase activity on a discrete analyser' (*Journal of Automatic Chemistry*, Vol. 4, No. 4, pp. 183–185). In it the author uses regression analysis to evaluate the similarity of results.

It is a common error on the part of all of us to ignore the basic premise in linear regression that the $x$ variable is taken to be the independent variable. As long as the correlation coefficient ($r$) is close to 1·00 the effect on the slope estimate $b_1$ is small. However, when $r = 0{\cdot}9$ the slope $b_2$ of the equation obtained by choosing $y$ to be the independent variable, rather than $x$, is 19% steeper than $b_1$. In fact $b_2$ is equal to $b_1/r^2$. Thus, since both lines pass through the point defined by the average of the two data-sets, the intercepts are affected as well.

So on the assumption that the manual method is the independent variable the author determined that the automated procedure yields results according to the equation:

$$y = 0{\cdot}90\,x + 0{\cdot}14 \qquad (r = 0{\cdot}87).$$

But if the more likely assumption had been made that the automated procedure was the independent variable, it can be calculated that the corresponding equation would have been:

$$y = 1{\cdot}19x - 0{\cdot}10.$$

The new slope and intercept are calculated from the correlation coefficient and the mean values of the two methods as stated in the paper.

| | | | |
|---|---|---|---|
| Thus | 1·19 | $= 0{\cdot}90/r^2$ | $(= b_2)$ |
| and | 0·10 | $= \bar{y} - 1{\cdot}19\bar{x}$ | |
| where | $\bar{y}$ | $= 0{\cdot}86$ and $\bar{x} = 0{\cdot}81$. | |

When one realizes that these two equations differ in slope by almost 30%, because of their dependence on the correlation coefficient, one begins to question the validity of conclusions drawn from the use of linear regression.

In the absence of any proof that one of the two methods is the independent variable, one must appreciate that the probable relationship lies somewhere between these two extreme positions. Since they both depend on $r$ it is interesting to speculate that this central equation is given by:

| | | |
|---|---|---|
| | $y = 1{\cdot}03x + 0{\cdot}02$ | |
| where | $1{\cdot}03 = 0{\cdot}90/0{\cdot}87$ | $(= b_0)$ |
| and | $0{\cdot}02 = \bar{y} - 1{\cdot}03\bar{x}$ | |
| note: | $b_0 = b_1/r = (b_1 \cdot b_2)^{1/2}$. | |

The equation has the slope that would obtain if $r$ was 1·00 rather than 0·87. Interestingly, it indicates that the two methods agree on average within 3% with almost no 'blank' bias. This is certainly a more encouraging finding than the original one which indicated compensating 'recovery' and 'blank' biases of −10% and +0·14 respectively.

A paper on this subject appeared in the proceedings of the 1976 8th Materials Research Symposium held at NBS in Gaithersburg, Maryland, USA. In it, techniques are proposed for verifying the validity of a linear regression equation [1].

The primary concern of the chemist is to obtain an estimate of the real relationship between methods. Linear regression is an extremely useful tool but its estimates are biased by statistical considerations. C. R. Milner *et al.*'s excellent paper would be even more convincing if the statistical bias of the equations had been recognized.

### Reference

1. King, D. E., in *Methods and Standards for Environmental Measurement* (NBS Special Publication 464, USGPO Washington, 1977).

*Environment Ontario, PO Box 213, 125 Resources Road, Rexdale, Ontario, Canada M9W 5L1*

---

*Correspondence from C. R. Milner, J. E. Buttery and B. R. Chamberlain*

Thank you for allowing us to respond to D. E. King's letter regarding the use of regression analysis. The manual transketolase method of Smeets is an established method, widely accepted since 1971, and is the method to which most others are referred. For this reason, the manual method was chosen as the independent variable.

The regression line equation was used to demonstrate the proportional relationship between the two techniques and is not intended to be used for the interconversion of results. The results obtained by the new method are interpreted from the reference range derived by the assay method.

However, we do take Dr King's point that neither method is a dependent variable and for this reason, the least squares regression is not the method of choice for the statistical analysis.

There are other methods of analysis that would be more suitable such as Deming's method which has the advantage in that it takes into account the random errors of both methods. Dr King's analysis of taking the central equation about the two regression lines agrees very well with Deming's procedure, which produces a slope of 1·03 and an intercept of 0·03.

In clinical chemistry there does not seem to be a common view as to the choice of a statistical method that should be used to evaluate the results. Unfortunately, the least squares linear regression is used by countless authors in numerous journals although it is not the most reliable method. Various articles have been written pointing out the error of using the linear regression analysis but their advice has not been heeded.

We thank Dr King for bringing his point of view to our attention and for demonstrating that our data produced a relationship between the two methods which is far better than we had at first realized.

*Department of Clinical Chemistry, The Queen Elizabeth Hospital, Woodville, South Australia 5011, Australia*

